# Predictive Modelling of Diabetes Risk in Population Groups Defined by Socioeconomic and Lifestyle Factors in Canada: A Cross-Sectional Study

**DOI:** 10.3389/ijph.2024.1607060

**Published:** 2024-08-20

**Authors:** Katherine Lu, Kathy Kornas, Laura C. Rosella

**Affiliations:** ^1^ Dalla Lana School of Public Health, University of Toronto, Toronto, ON, Canada; ^2^ ICES, Toronto, ON, Canada; ^3^Institute for Better Health, Trillium Health Partners, Mississauga, ON, Canada; ^4^ Department of Laboratory Medicine and Pathobiology, Temerty Faculty of Medicine, University of Toronto, Toronto, ON, Canada

**Keywords:** type 2 diabetes, equity, prediction model, population health, prevention

## Abstract

**Objectives:**

This study modelled diabetes risk for population groups in Canada defined by socioeconomic and lifestyle characteristics and investigated inequities in diabetes risk using a validated population risk prediction algorithm.

**Methods:**

We defined population groups, informed by determinants of health frameworks. We applied the Diabetes Population Risk Tool (DPoRT) to 2017/2018 Canadian Community Health Survey data to predict 10-year diabetes risk and cases across population groups. We modelled a preventive intervention scenario to estimate reductions in diabetes for population groups and impacts on the inequity in diabetes risk across income and education.

**Results:**

The population group with at least one lifestyle and at least one socioeconomic/structural risk factor had the highest estimated 10-year diabetes risk and number of new cases. When an intervention with a 5% relative risk reduction was modelled for this population group, diabetes risk decreased by 0.5% (females) and 0.7% (males) and the inequity in diabetes risk across income and education levels was reduced.

**Conclusion:**

Preventative interventions that address socioeconomic and structural risk factors have potential to reduce inequities in diabetes risk and overall diabetes burden.

## Introduction

Diabetes is a chronic disease that affects hundreds of millions worldwide, and is attributed to an estimated 2 million deaths globally in 2019 [1]. In Canada, type 2 diabetes is a large and growing health issue, impacting an estimated 5.7 million people in 2022 [2]. Treating diabetes and related conditions cost the Canadian healthcare system an estimated 15.36 billion CAD between 2011 and 2022 [3].

Diabetes disproportionately impacts certain populations, representing a health equity issue. For instance, diabetes incidence and prevalence are greater in populations with lower income compared to higher income groups [4, 5]. Diabetes prevalence and incidence have also been found to be higher in many racialized populations [6]. Given that diabetes risk and burden vary across population groups, identifying high-risk populations is important for diabetes prevention planning.

Population health assessment activities involve assessing the complete distribution of risk in populations, identifying priority populations for intervention, and identifying effective strategies that address risk distribution and health inequities. Two common approaches for identifying priority populations are the health equity approach and the burden of disease approach [7]. The health equity approach focuses on identifying disadvantaged populations with high risk, while the burden of disease approach focuses on identifying population groups based on their relative contribution to the total disease burden in the population. Proportionate universalism asserts that interventions should be applied across the entire population at levels of action proportional to the needs of population subgroups [7]. Modelling approaches provide a practical guide to understand diabetes risk across population groups [8] and identify how to execute proportionate universalism.

Population-based predictive risk algorithms are practical tools for population health assessment and intervention modelling, and can inform how to consider both health equity and disease burden considerations [9]. The Diabetes Population Risk Tool (DPoRT) is a validated algorithm that predicts 10-year incidence of physician-diagnosed diabetes using population survey data [10]. This tool is suited for applying an equity lens to diabetes prevention planning. First, DPoRT estimates the distribution of diabetes risk and the expected number of cases in the population across population subgroups. Second, DPoRT can model diabetes prevention scenarios to estimate population benefits for priority population groups.

This study aimed to demonstrate how to explicitly include equity considerations in determining priority populations for diabetes prevention planning. Our objectives were to use a health determinants framework and apply DPoRT to the population living in Canada to identify high-risk populations for Type 2 diabetes and estimate inequities in diabetes risk. We also modelled the population benefit of a diabetes prevention intervention scenario targeted to population groups defined by socioeconomic/structural and lifestyle factors on reducing inequities in diabetes risk.

## Methods

### Data Source and Study Population

Data from the 2017 to 2018 Canadian Community Health Survey (CCHS) public use microdata file (PUMF) was used [11]. Briefly, the CCHS is a national cross-sectional survey that collects information related to health. The CCHS uses a multi-stage sample allocation strategy, collects data from Canadians 12 years and over, and samples respondents over each 2 year period [12]. The sampling frame excludes people living on reserves and First Nations settlements, institutionalized populations, and full-time members of the Canadian Forces. The CCHS uses an area frame to sample respondents 18 years and over. The methodology of the CCHS is described in detail elsewhere [12]. Our study population includes respondents from all ten provinces in Canada, aged 20 years and over, not pregnant, and with no self-reported diagnosis of diabetes at the time of interview. Respondents from the Territories were excluded because information on household income was not collected, which is a required variable for the DPoRT risk algorithm used in this study.

### Guiding Framework

**FIGURE 1:**
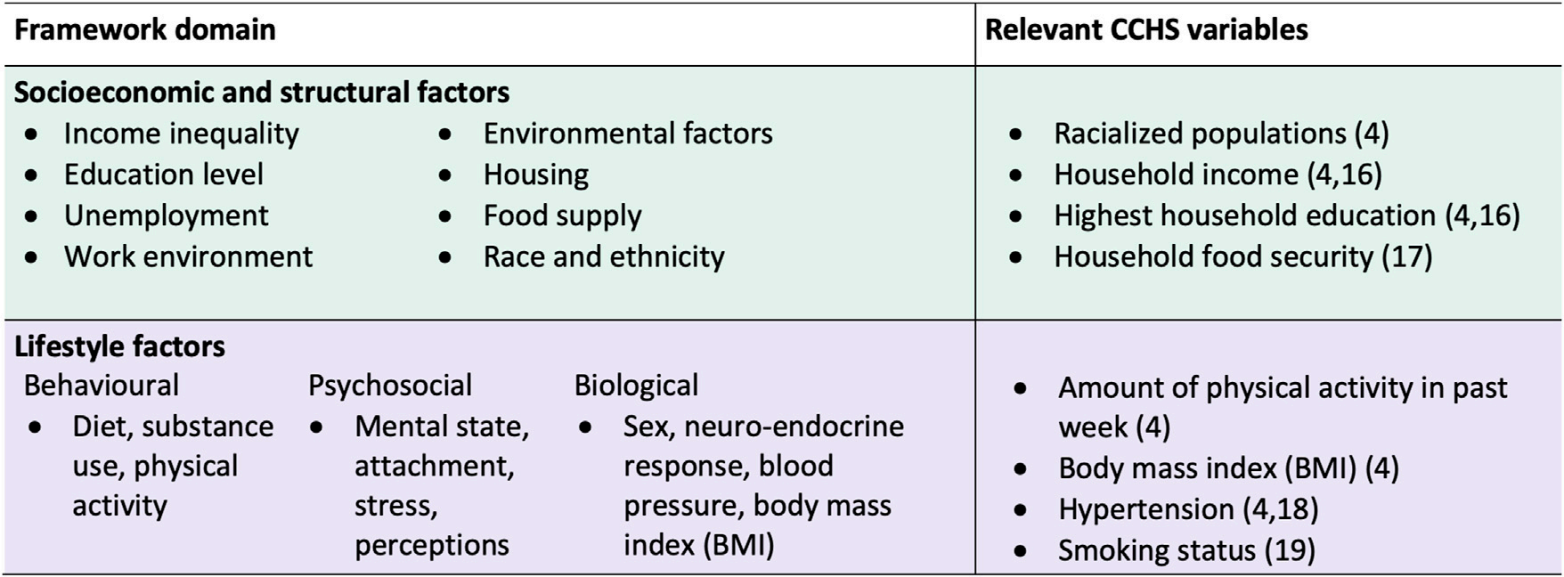
Conceptual framework to guide selection of health determinants to measure population inequities in diabetes risk, and corresponding 2017/18 Canadian Community Health Survey variables. Framework synthesized from the Wider Determinants of Health Model and the Queensland Health Framework for Addressing the Social Determinants of Health and Wellbeing (Canada. 2017/18).

We developed a conceptual framework to apply a health equity lens to the selection of health determinants for our analysis (Figure 1). The Wider Determinants of Health Model was used as a base [13]. The Wider Determinants Model is commonly used to identify different levels of factors that affect health, including socioeconomic and structural factors, social and community networks, and individual lifestyle behaviours [14]. This model is also used by the 2018 Ontario Health Equity Guideline for public health units, demonstrating its applicability to the Canadian context [15]. To build on the broad framework of the Wider Determinants Model, we integrated factors identified in the Queensland Health’s Framework for Addressing the Social Determinants of Health and Wellbeing [16]. The Queensland Health Framework was chosen for its applicability to the general population, its overlap in themes with the Wider Determinants model, and its detailed inclusion of specific health factors. The two frameworks were combined by identifying common domains and health factors in each framework: 1) socioeconomic and structural (macro-level) and 2) individual lifestyle (micro-level).

### Variable Selection

Relevant variables from the survey were selected for investigation based on the factors within each domain of our framework (Figure 1). We chose variables that have established associations with diabetes risk and for which data was collected from all provinces (Figure 1).

For the socioeconomic and structural domain, we included the following CCHS variables: racialized population (yes, no); household income quintile (lowest, low-middle, middle, high-middle, and highest); household education (less than secondary, secondary, and post-secondary); and household food insecurity (severely food insecure, moderately food insecure, food secure). Racialized population was used to refer to the visible minority concept defined in the CCHS. Visible minority, as defined by the Employment Equity Act, refers to an individual’s membership in a visible minority group, and includes people who are non-Caucasian in race or non-white in colour. The racialized population consists mainly of the following groups: South Asian, Chinese, Black, Filipino, Arab, Latin American, Southeast Asian, West Asian, Korean, Japanese, and Aboriginal Identity. Household income quintiles were created by Statistics Canada based on the value for the adjusted ratio of their household income to the low income cut-off corresponding to their household and community size [12]. Being part of a racialized group, having lower household income, having lower education, and being food insecure have been found to be associated with diabetes risk [4, 17, 18].

For the lifestyle factors domain, we included: physical activity (PA) measured in metabolic equivalents of task (METs) (PA = 0, 0 < PA < 450, 450 ≤ PA < 900, and PA ≥ 900 METs*min/week); body mass index (BMI) (BMI < 23, 23 ≤ BMI < 25, 25 ≤ BMI < 30, 30 ≤ BMI < 35, and 35 ≥ BMI kg/m^2^); high blood pressure (yes, no); and current smoking status (yes, no) [19, 20] (Figure 3). Having low levels of physical activity, high BMI, high blood pressure, and smoking have been shown to increase the risk of developing diabetes [4, 21, 22].

The following health determinants were considered for the analysis, but were excluded due to data availability limitations of the 2017/18 CCHS: employment/occupation group (skipped for 39.5% of survey participants), housing stability (no relevant variables), social support/networks/capital (only asked for Alberta and British Columbia participants), diet/fruit and vegetable consumption (only asked for participants from the Territories), access to healthcare services (skipped for 41.3% of participants due to only being asked for some provinces). We also excluded subjective variables such as work satisfaction and self-rated mental health.

### Population Groups

**FIGURE 2:**
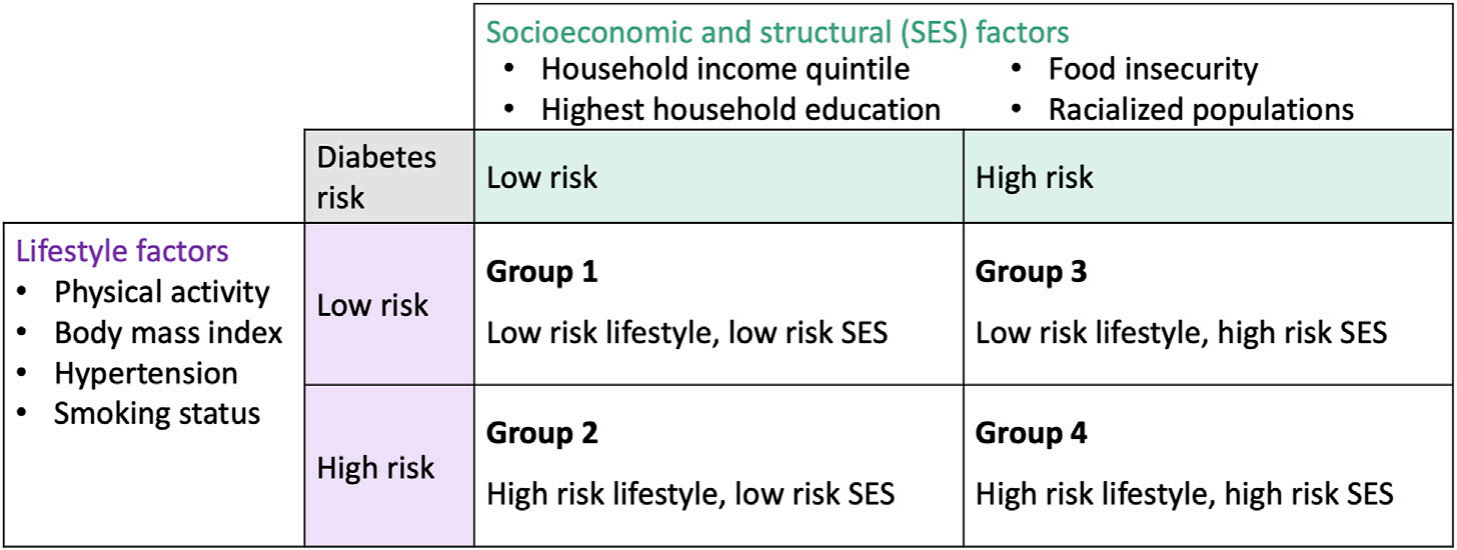
Population groups based on the conceptual framework and 2017/18 Canadian Community Health Survey variables (Canada. 2017/18).

To examine population inequities in diabetes risk, we defined population groups according to the socioeconomic/structural and lifestyle domains of our framework. Respondents were classified into four population groups based on the presence of risk factors within the framework domains (Figure 2). Respondents were classified as having high risk for a domain if they had an unfavourable level of a risk factor for at least one of the variables within that domain. Conversely, respondents were classified as having low risk for a domain if they had favourable levels of a risk factor for all of the variables within that domain. Levels within each variable were determined to be favourable (i.e., associated with low diabetes risk) or unfavourable (i.e., associated with high diabetes risk) based on previous literature. Definitions for variable-level risk classification can be referred to in Supplementary Table S1. We note that we classified both overweight (25 kg/m^2^ ≤ BMI < 30 kg/m^2^) and obese (BMI ≥ 30 kg/m^2^) categories as unfavourable levels (i.e., high risk for diabetes) since both overweight and obese BMI have been linked to greater diabetes risk [23].

### Estimating Diabetes Risk

The Diabetes Population Risk Tool (DPoRT) is a validated population-based risk prediction algorithm that estimates future incidence of physician-diagnosed type 2 diabetes (T2D) for a 10-year period [10]. DPoRT uses a statistical model based on the Weibull Survival distribution. The original risk algorithm was developed based on a cohort of 19,861 individuals without diabetes followed between 1996 and 2005, and was validated in two external cohorts in the provinces of Ontario (n = 26,465) and Manitoba (n = 9,899), as well as across ethnic groups [10, 24, 25]. The cohorts linked baseline risk factors to a validated population-based diabetes registry to ascertain diabetes diagnosis during follow-up. The DPoRT model coefficients were updated in a more recent Ontario cohort (n = 69,606), with follow-up until 2011 [24]. The updated DPoRT model has demonstrated high overall predictive performance, good discrimination (C = 0.77) and calibration (H-L X2 = <20) [24]. DPoRT uses sex-specific models with the following predictors: age, BMI, ethnicity, education, immigrant status, prior diagnosis of hypertension, prior heart disease, household income quintile, and smoking. The details of the development and validation of DPoRT are described elsewhere [10, 24]. The DPoRT algorithm is included in Supplementary Figure S2. In the DPoRT model for females, missing BMI is included as a predictor because it was found to be predictive of diabetes risk and its inclusion improved model performance [10].

### Statistical Analysis

We applied DPoRT to our study cohort to predict incident diabetes risk and cases for the 10-year period of 2017/18 to 2027/28. Individuals with missing risk factor information required for the DPoRT algorithm were excluded (n = 4,816) (Supplementary Figure S1). The total proportion of missing in our dataset was 5.3%. The amount of missing for any one predictor variable required for DPoRT ranged from 0.2% to 2.2%. The excluded group with missing predictor information were more likely to be male and had a larger proportion of individuals ≥65 years old and low income, compared to the study cohort (Supplementary Table S3). In addition, we conducted a sensitivity analysis where each missing variable in the predictive model was assigned the most frequent category, as recommended by Harrell [26], to examine the impact of missing data. Diabetes risk estimates were averaged across all respondents of the study population to determine the population-level risk of diabetes. The baseline 10-year DPoRT risk and number of new cases were estimated across variables identified in our framework and for each population group. The number of new (incident) cases of diabetes was estimated by multiplying the average risk by the population size, using sampling weights provided by Statistics Canada. Results were stratified by sex because there are sex differences in diabetes risk factors [27].

The CCHS uses self-reported height and weight to calculate BMI. To account for reporting errors in self-reported height and weight, we used the equations developed by Statistics Canada. The details of the derivation of these equations are explained elsewhere [28].

We developed a diabetes prevention intervention scenario to model the impact of targeting population groups defined by lifestyle and socioeconomic/structural risk factors. The target groups for modelling consisted of the population groups defined in Figure 2. First, we used DPoRT to estimate the baseline 10-year diabetes risk for each population group. Then we applied a relative risk reduction of 5% to the baseline diabetes risk of each target group. The relative risk reduction was chosen for a modest effect expected to be achievable in population-level interventions [29]. We calculated the absolute reduction in diabetes risk (absolute difference of diabetes risk at baseline and diabetes risk after the scenario) and the number of new diabetes cases prevented (absolute difference of diabetes cases at baseline and diabetes cases after the scenario).

We modelled inequities in diabetes risk, which we defined as a health difference that adversely affects disadvantaged populations on the basis of higher diabetes risk. We used DPoRT to estimate diabetes risk across income quintiles and household education levels at baseline and post-intervention scenario. We calculated the difference in diabetes risk between the most and least disadvantaged income and education groups at baseline and after the scenario.

We also conducted a sensitivity analysis to examine the impact of a different risk threshold in defining the population groups on the modelling results. In this sensitivity analysis, population groups were defined as follows: group 1 (≤1 high risk factor in the lifestyle domain and 0 risk factors in the socioeconomic/structural domain); group 2 (at least two high-risk factors in the lifestyle domain and 0 risk factors in the socioeconomic/structural domain); group 3 (≤1 high risk factor in the lifestyle domain and at least one high-risk factor in socioeconomic/structural domain); group 4 (at least two high-risk factors in the lifestyle domain and at least one high risk factor in socioeconomic/structural domain).

All analysis was conducted using SAS version 9.4 (SAS Institute, Cary, NC, United States).

## Results

A total of 113,290 participants were included in the 2017/18 CCHS. After applying exclusion criteria, our study cohort consisted of 85,706 respondents (Supplementary Figure S1).

Table 1 shows baseline characteristics and DPoRT estimated number of new diabetes cases for the study cohort. The cohort included similar proportions of each sex with 52.7% females. Overall, most people had at least secondary education and were food secure. A large proportion of the sample was overweight (BMI 25.0–29.9 kg/m^2^) or obese (BMI ≥ 30.0 kg/m^2^). Most of the sample did not have hypertension or were not current smokers, and the majority had weekly physical activity levels above 450 METs.

**TABLE 1:** Baseline characteristics at survey date and DPoRT estimated 10-year diabetes risk and case estimates for Canada 2017/18 to 2027/2028 (Canada. 2017/18).

	Proportion of population (%)	10-year diabetes risk (%)	Number of new cases (1000s)
Overall (n = 85,706, represented population = 24,244,479)	100.0	9.9	2397.1
Sex			
Female	52.7	8.6	1097.4
Male	47.3	11.3	1299.7
Age group (years)			
Age ≤ 45	46.5	4.9	549.2
45 < Age < 65	35.0	13.7	1166.0
Age ≥ 65	18.5	15.2	681.9
Province/Territory			
Newfoundland and Labrador	1.5	9.9	35.2
Prince Edward Island	0.4	9.5	9.4
Nova Scotia	2.6	9.9	63.1
New Brunswick	1.9	9.8	47.2
Quebec	23.6	9.8	560.6
Ontario	38.3	9.9	919.9
Manitoba	3.4	9.8	80.5
Saskatchewan	3.0	9.6	68.8
Alberta	11.7	10.0	284.5
British Columbia	13.5	9.9	327.8
Racialized population group			
Yes	25.5	12.1	747.5
No	74.4	9.1	1649.5
Household income quintile			
Lowest	18.7	10.5	476.4
Low-middle	19.2	10.6	491.4
Middle	20.0	10.0	489.2
High-middle	20.7	9.7	487.6
Highest	21.4	8.7	451.8
Highest household education			
Less than secondary	9.9	15.2	368.6
Secondary graduation	23.0	11.5	638.7
Post-secondary	67.0	8.6	1389.7
Household food insecurity			
Severely food insecure	2.4	9.9	57.9
Moderately food insecure	5.2	10.6	134.2
Food secure	91.7	9.8	2186.6
Amount of physical activity (PA) in past week (METs*min/week)			
PA = 0	19.1	12.4	575.4
0 < PA < 450	20.6	10.4	520.4
450 ≤ PA < 900	16.9	9.3	381.2
PA ≥ 900	41.1	8.6	855.6
BMI (Body mass index, kg/m^2^)			
BMI < 23	21.6	3.1	162.8
23 ≤ BMI < 25	16.4	5.3	209.1
25 ≤ BMI < 30	36.7	9.7	862.0
30 ≤ BMI < 35	14.4	17.7	615.8
BMI ≥ 35	6.8	24.4	404.1
Hypertension			
Yes	16.0	20.9	812.4
No	84.0	7.8	1584.6
Current smoker			
Yes	17.1	9.2	380.5
No	82.9	10.0	2011.8

The DPoRT estimated 10-year diabetes risk for Canada was 9.9%, which corresponds to an estimated 2.4 million new diabetes cases. DPoRT estimated diabetes risk was higher for males at 11.3% compared to females at 8.6%. There were differences in diabetes risk across population groups, including higher estimated risk for older age groups, racialized groups, low-income groups, low education levels, and individuals with high BMI. We conducted a sensitivity analysis to examine the impact of missing data. The results of the sensitivity analysis and the results presented did not differ substantively (see Supplementary Table S4).

The weighted baseline characteristics stratified by the four population groups (defined by lifestyle and socioeconomic characteristics) is shown in Supplementary Table S2. Population groups 1, 2, 3, and 4 represented 8.1%, 27.9%, 11.4%, and 53.5% of the population, respectively. Population group 1 was largely composed of females (66.6%) and the younger age group (≤45 years old) (54.7%). Population group 1 had a large proportion of individuals with more favourable lifestyle and SES factors [e.g., non-racialized group (100.0%), highest income quintile (42.4%), BMI 23–25 (42.6%)]. Population group 2 was more represented by males (53.3%), younger age (≤45) and middle age (45–65) groups, 40.3% and 42.5%, respectively. Population group 2 had a large proportion of individuals with favourable SES factors [e.g., non-racialized group (100.0%), highest income quintile (39.5%)] and unfavourable lifestyle factors [e.g., BMI 25–30 (50.3%)]. Population group 3 had a large proportion of females (62.9%) and young age group, ≤45 years old (62.9%). Population group 3 had a large proportion of individuals with unfavourable SES factors [racialized group (48.1%), lowest income quintile (28.7%)] and favourable lifestyle factors [e.g., BMI 23–25 (54.5%)]. Population group 4 had a similar gender distribution [females (48.6%)], and large proportion of the younger age group, ≤45 (44.6%). Population group 4 had a large proportion of individuals with unfavourable SES factors [e.g., low income (28.7%)] and unfavourable lifestyle factors [e.g., BMI 25–30 (43.2%)].

**FIGURE 3:**
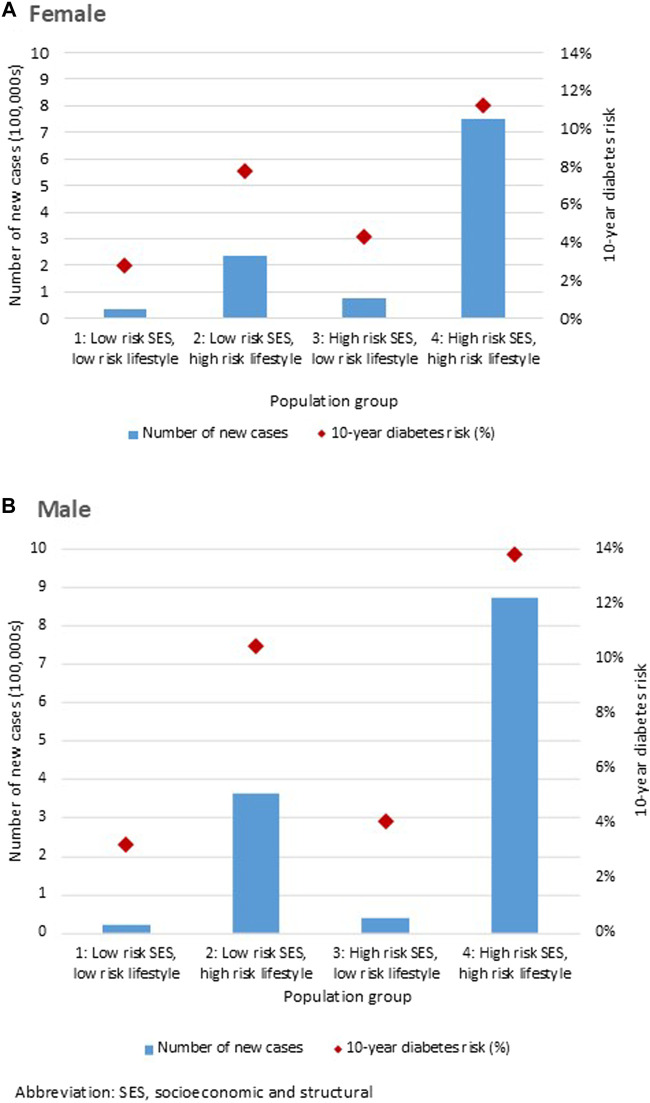
Diabetes Population Risk Tool diabetes risk and case estimates for Canada 2017/18 to 2027/28, by population groups, for **(A)** females and **(B)** males (Canada. 2017/18).

Figure 3 shows the DPoRT 10-year diabetes risk and number of new cases by the population groups, stratified by sex. Predicted 10-year diabetes risk and cases were greater for the population group with at least one high-risk factor in the lifestyle domain (group 2) or socioeconomic/structural domain (group 3), compared to the population group without high-risk factors (group 1). The fourth population group (group 4), consisting of the population with at least one risk factor in both the socioeconomic/structural and lifestyle domains, had the highest estimated diabetes risk and the largest number of future diabetes cases. The results were similar between males and females.

We modelled an intervention scenario (5% relative risk reduction) targeted to the population groups. Table 2 shows the absolute reduction in diabetes risk and number of cases averted for each population group. We estimated that an intervention targeted to the population with at least one high-risk factor in the socioeconomic and structural domain (group 3) would reduce risk by 0.2% among females and males. An intervention targeted to the population with at least one high-risk factor in the lifestyle domain (group 2) would reduce risk by 0.4% in females and 0.5% in males. The largest reduction was estimated with an intervention targeted to the population with at least one high-risk factor in both the socioeconomic/structural and lifestyle domains (group 4) in which diabetes risk decreased by 0.5% in females (37,400 cases averted) and 0.7% in males (43,600 cases averted).

**TABLE 2:** Diabetes Population Risk Tool (DPoRT) 10-year risk reductions and cases averted for Canada 2017/18 to 2027/28 at baseline and with a hypothetical intervention scenario that applies a 5% diabetes relative risk reduction (Canada. 2017/18).

	Health determinant domain		10-year diabetes risk			Number of new diabetes cases (thousands)		
Group	SES^a^	Lifestyle	Baseline (%)	5% relative risk reduction	Absolute risk difference from baseline (%)	Baseline (1000s)	5% relative risk reduction	Cases averted (1000s)
Overall								
1	Low risk	Low risk	2.9	2.7%	0.2	57.5	54.6	2.9
2	Low risk	High risk	9.2	8.7%	0.5	601.8	571.7	30.1
3	High risk	Low risk	4.2	4.0%	0.2	117.0	111.1	5.9
4	High risk	High risk	12.5	11.9%	0.6	1620.9	1539.9	81.0
Females								
1	Low risk	Low risk	2.8	2.6%	0.2	36.3	34.5	1.8
2	Low risk	High risk	7.8	7.4%	0.4	236.8	224.9	11.9
3	High risk	Low risk	4.3	4.1%	0.2	75.2	71.4	3.8
4	High risk	High risk	11.2	10.7%	0.5	749.1	711.7	37.4
Males								
1	Low risk	Low risk	3.2	3.1%	0.2	21.1	20.1	1.0
2	Low risk	High risk	10.5	10.0%	0.5	365.0	346.7	18.3
3	High risk	Low risk	4.1	3.9%	0.2	41.8	39.7	2.1
4	High risk	High risk	13.8	13.1%	0.7	871.8	828.2	43.6

^a^ SES, socioeconomic/structural.

We estimated the effect of the intervention scenario on reducing the inequity in diabetes risk across income and education levels (Table 3). We defined inequity as the difference in diabetes risk between the highest and lowest risk groups. At baseline, the inequity, or absolute difference in diabetes risk was 1.8%, comparing the lowest and highest income quintiles (Table 3). The absolute difference in diabetes risk did not decrease with an intervention targeted to the population with atleast one high risk factor in the socioeconomic/structural domain (group 3). The absolute difference in diabetes risk decreased to 1.5% with an intervention targeted to the population defined by having atleast one high risk factor in both socioeconomic/structural and lifestyle domains (group 4). Conversely, the absolute difference in diabetes risk increased to 2.0% with an intervention targeted to the population with atleast one high risk factor in the lifestyle domain (group 2). The results were similar for diabetes inequity across education levels. The absolute difference in diabetes risk was 6.6% at baseline, comparing the most and least educated groups. Post-intervention, the absolute difference in diabetes risk comparing the lowest and highest education levels was 6.8%, 6.7%, and 6.2% when targeting composite risk groups 2, 3, and 4, respectively.

**TABLE 3:** Diabetes Population Risk Tool (DPoRT) risk estimates and inequity across income quintiles and household education levels for Canada 2017/18 to 2027/28, before and after an intervention that results in a 5% relative risk reduction (Canada. 2017/18).

Income quintile	Baseline	After intervention^c^		
		Target population group: 2	Target population group: 3	Target population group: 4
Lowest (1)	10.5%	10.5%	10.5%	10.0%
Low-middle (2)	10.6%	10.6%	10.5%	10.1%
Middle (3)	10.0%	9.9%	10.1%	9.8%
High-middle (4)	9.7%	9.5%	9.7%	9.5%
Highest (5)	8.7%	8.5%	8.7%	8.5%
Inequity Q5−Q1 (absolute)^a^	1.8%	2.0%	1.8%	1.5%
Inequity (relative)^b^	17.0	19.0	17.1	15.0
Household education				
Less than secondary (1)	15.2%	15.2%	15.2%	14.5%
Secondary graduation (2)	11.5%	11.5%	11.4%	10.9%
Post-secondary (3)	8.6%	8.4%	8.5%	8.3%
Inequity 1–3 (absolute)^a^	6.6%	6.8%	6.7%	6.2%
Inequity (relative)^b^	43.4	44.7	44.1	42.8

^a^ Absolute inequity: risk difference between the most and least disadvantaged groups.

^b^ Relative inequity: risk difference between the most and least disadvantaged groups, divided by the risk of the most disadvantaged group.

^c^ Interventions were applied to population group 2 (at least one high-risk factor in lifestyle domain), group 3 (at least one high-risk factor in socioeconomic/structural domain), or group 4 (at least one high-risk factor in both lifestyle and socioeconomic/structural domains).

We conducted a sensitivity analysis to examine the impact of a different risk threshold for the population groups. In this analysis, population groups 1, 2, 3, and 4 represented 23.6%, 11.5%, 37.0% and 27.9% of the population, respectively. The results of the sensitivity analysis for the intervention scenarios showed a similar pattern for the absolute risk difference from baseline and for the number of cases averted (i.e., population group 4 had the most cases averted) (see Supplementary Table S5). The results for the difference in the inequity in diabetes risk across income and education levels was also similar (see Supplementary Table S6).

## Discussion

This study identified priority populations and measured equity gaps in future diabetes risk in Canada. Our results estimated higher 10-year diabetes risk for populations with at least one lifestyle risk factor and at least one socioeconomic/structural risk factor. Intervention scenario modelling suggested that the inequity gap in diabetes risk would narrow with a preventative intervention targeted to the population group with both socioeconomic and lifestyle risk factors. We found that the most equitable preventative intervention would be a multifactorial approach that could address both socioeconomic and lifestyle risk factors.

Our method for defining population groups combines the health equity and burden of disease approaches that are commonly used to identify priority groups in public health [7]. By applying a health determinants framework and estimating risk using DPoRT, we could look at high risk populations across a range of social determinants. By including variables known to be associated with diabetes and estimating future diabetes risk and number of new cases, we were able to explore populations with disproportionate burden of disease. Our study identified the population group with both lifestyle and socioeconomic/structural risk factors as both the highest risk and highest burden group, indicating this population as high priority. Diabetes risk was also elevated for the population groups with lifestyle risk factors only and socioeconomic/structural risk factors only.

Our study adds to the literature by predicting future trends of diabetes risk from the combined effect of both lifestyle and socioeconomic/structural factors, for the Canadian population. Many existing studies have demonstrated the associations between diabetes and the individual lifestyle and socioeconomic risk factors in our analysis. In particular, a 2018 review on risk factors and prevention of Type 2 diabetes in Canada synthesized the role of ethnicity, obesity, socioeconomic status, among other factors [23]. Relative to SES, a study using data from the Canadian National Population Health Survey found an association between low income and diabetes incidence after adjusting for factors including age, obesity, and psychological distress [30]. Another study using 2000–2008 CCHS data found a strong association between income and Type 2 diabetes prevalence, also considering many factors such as hypertension, racialized status, and obesity [4]. Our study illustrates an approach for investigating the combined effect of diabetes-relevant risk factors on future diabetes incidence in the population to support preventative population health planning.

Our scenario modelling results suggested that an intervention targeted to population groups defined by socioeconomic and lifestyle characteristics would be successful in reducing diabetes risk overall, but would have varying success in reducing inequities in diabetes risk across income and education levels. We found that an intervention that focuses on people in both the high-risk SES and lifestyle risk group would both reduce future diabetes risk and meaningfully narrow inequity gaps in diabetes risk between the lowest and highest income and education levels. Historically, much of diabetes prevention has focused on lifestyle modifications. For example, many landmark trials, such as the Diabetes Prevention Program, consists of lifestyle interventions (e.g., to improve physical activity and diet) and pharmacotherapies like metformin [23, 31]. The 12-month long Canadian Diabetes Prevention Program also focused on lifestyle changes such as weight loss [2]. To reduce inequity in diabetes risk, future programs should incorporate interventions that also target socioeconomic and structural health determinants. The new 2022 Framework for Diabetes in Canada published by the Public Health Agency of Canada (PHAC) is a step in the right direction and describes multilevel factors for the prevention of diabetes [32]. PHAC suggests interventions that require less individual agency, such as implementing physical activity and healthy diets in schools. Other potential interventions include changing the built environment to improve walkability for high-risk populations [33].

The study results should be interpreted in the context of the study limitations. Firstly, our analysis does not account for health determinants at the meso-level (i.e., factors related to community contexts and healthcare/organizational settings) due to the unavailability of this data in the 2017/18 CCHS. For example, community belonging was the only CCHS variable identified that was related to the “community and social context” and which fit our criteria. Similarly, the only variable related to the factors under “healthcare” that fit our criteria was having a regular healthcare provider. Because a single variable cannot adequately represent these domains, we concentrated our analysis on the socioeconomic/structural and lifestyle domains. Within the socioeconomic/structural and lifestyle domains, we were unable to include some variables in our analysis, such as diet, due to these data only being collected for one or a few provinces. Future research could apply data linkage to address factors missing in this study. Despite CCHS limitations, our analysis still covers the important social determinants of diabetes, such as education, income, and food security [34].

Although the sampling frame of the CCHS is representative of 98% of the Canadian population, some population groups are excluded, including full-time members of the Canadian Armed Forces, institutionalized individuals and individuals living on Indigenous reserves, who are known to be disproportionately impacted by diabetes [12, 35]. We also excluded individuals living in the territories from our analysis due to missing income data, which is required for the DPoRT risk algorithm. Thus, the results may not be generalizable to these populations. The CCHS also relies on self-reported data, which can be prone to misclassification. However, to account for some error, we applied a correction equation for self-reported BMI [28]. Finally, we chose to not apply age-standardization to the risk estimates for population groups given that age-standardization may not convey the true magnitude of risks or inequities in the population [36]. However, the risk estimates are adjusted for age since age is included as a predictor in the DPoRT model.

### Conclusion

In this study, we applied a validated diabetes population risk prediction tool to identify priority populations for diabetes prevention from both a health equity and disease burden perspective. These findings suggest that populations with risk factors that overlap in the socioeconomic/structural and lifestyle domains are an important target group. Future diabetes preventative programming and policy can consider a multilevel lens that addresses socioeconomic/structural and lifestyle risk factors to potentially narrow the inequity gap in diabetes risk.
