# Supplementary material for: Predictive Modelling of Diabetes Risk in Population Groups Defined by Socioeconomic and Lifestyle Factors in Canada: A Cross-Sectional Study
Source: Int J Public Health. 2024 Aug 20;69:1607060. doi: 10.3389/ijph.2024.1607060 (PMC11368776; doi:10.3389/ijph.2024.1607060)
Supplement: Supplementary file 1 [file DataSheet1.docx]

**SUPPLEMENTARY MATERIAL**

**Supplementary Figure S1.** 2017/2018 Canadian Community Health Survey (CCHS) cohort inclusion and exclusion criteria (Canada. 2017/18).


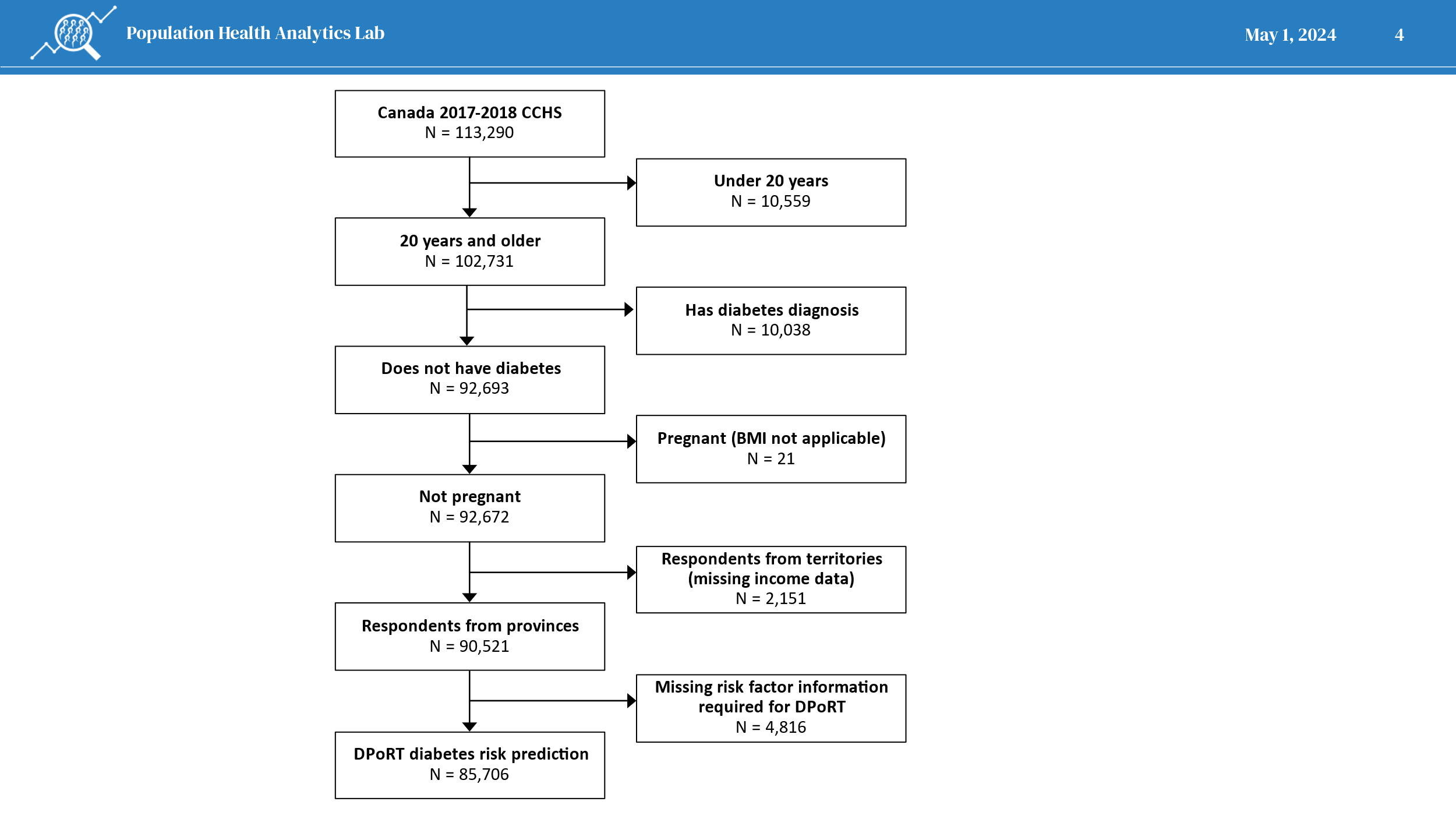


*Abbreviations: BMI, Body mass index; DPoRT, Diabetes Population Risk Tool*

**Supplementary Table S1.** Health determinants variables risk classification with Diabetes Population Risk Tool (DPoRT) risk and case estimates for Canada 2017/18 to 2027/28 (Canada. 2017/18).

| **Framework domain** | **Risk factor**  *Red/italicized = high risk* | **Proportion of population** | **10-year diabetes risk** | **Number of new cases (1000s)** |
| --- | --- | --- | --- | --- |
| Socioeconomic and structural | **Racialized population group** |  |  |  |
|  | *Yes* | 25.5% | 12.1% | 747.5 |
|  | No | 74.4% | 9.1% | 1649.5 |
|  | **Household income quintile** |  |  |  |
|  | *Lowest* | 18.7% | 10.5% | 476.4 |
|  | *Low-middle* | 19.2% | 10.6% | 491.4 |
|  | Middle | 20.0% | 10.0% | 489.2 |
|  | High-middle | 20.7% | 9.7% | 487.6 |
|  | Highest | 21.4% | 8.7% | 451.8 |
|  | **Highest household education** |  |  |  |
|  | *Less than secondary* | 9.9% | 15.2% | 368.6 |
|  | *Secondary graduation* | 23.0% | 11.5% | 638.7 |
|  | Post-secondary | 67.0% | 8.6% | 1389.7 |
|  | **Household food insecurity** |  |  |  |
|  | *Severely food insecure* | 2.4% | 9.9% | 57.9 |
|  | *Moderately food insecure* | 5.2% | 10.6% | 134.2 |
|  | Food secure | 91.7% | 9.8% | 2186.6 |
| Lifestyle | **Volume of physical activity in past week (METs*min/week)** |  |  |  |
|  | *PA = 0* | 19.1% | 12.4% | 575.4 |
|  | *0 < PA < 450* | 20.6% | 10.4% | 520.4 |
|  | 450 ≤ PA < 900 | 16.9% | 9.3% | 381.2 |
|  | 900 ≤ PA | 41.1% | 8.6% | 855.6 |
|  | **Body mass index (BMI)** |  |  |  |
|  | BMI < 23 | 21.6% | 3.1% | 162.8 |
|  | 23 ≤ BMI < 25 | 16.4% | 5.3% | 209.1 |
|  | *25 ≤ BMI < 30* | 36.7% | 9.7% | 862.0 |
|  | *30 ≤ BMI < 35* | 14.4% | 17.7% | 615.8 |
|  | *35 ≤ BMI* | 6.8% | 24.4% | 404.1 |
|  | **Hypertension** |  |  |  |
|  | *Yes* | 16.0% | 20.9% | 812.4 |
|  | No | 84.0% | 7.8% | 1584.6 |
|  | **Current smoker** |  |  |  |
|  | *Yes* | 17.1% | 9.2% | 2011.8 |
|  | No | 82.9% | 10.0% | 380.5 |

*Abbreviation: MET, Metabolic equivalent of task*

**Supplementary Figure S2.** Diabetes Population Risk Tool (DPoRT) Algorithm^1^.

**Males:**

μ = 10.3062

 − 0.3629 × hypertension

 − 0.3483 × heart disease

− 0.5697 × non-white ethnicity

− 0.0585 × smoker

+ 0.1884 × attended post-secondary

+ 0.1173 × top income quintile

− 0 × (BMI < 23 & age < 45)

− 0.5520 × (23 ≤ BMI < 25 & age < 45)

− 0.9521 × (25 ≤ BMI < 30 & age < 45)

− 1.7162 × (30 ≤ BMI < 35 & age < 45)

− 2.3310 × (35 ≤ BMI & age < 45)

− 1.3602 × (BMI < 23 & age ≥ 45)

− 1.6537 × (23 ≤ BMI < 25 & age ≥ 45)

− 2.0563 × 25 ≤ (BMI < 30 & age ≥ 45)

− 2.5513 × (30 ≤ BMI < 35 & age ≥ 45)

− 2.9353 × (35 ≤ BMI & age ≥ 45).

Scale = 0.7994

**Females:**

μ = 10.5777

− 0.4098 × hypertension

− 0.4528 × non-white ethnicity

− 0.1477 × immigrant

+ 0.1939 × attended post-secondary

− 0 × (BMI < 23 & age < 45)

− 0.7432 × (23 ≤ BMI < 25 & age < 45)

− 1.1521 × (25 ≤ BMI < 30 & age < 45)

− 1.8479 × (30 ≤ BMI < 35 & age < 45)

− 2.0562 × (35 ≤ BMI & age < 45)

− 1.5832 × (BMI = missing & age < 45)

− 0.7100 × (BMI < 23 & 45 ≤ age < 65)

− 1.2338 × (23 ≤ BMI < 25 & 45 ≤ age < 65)

− 1.8357 × (25 ≤ BMI < 30 & 45 ≤ age < 65)

− 2.3742 × (30 ≤ BMI < 35 & 45 ≤ age < 65)

− 2.6631 × (35 ≤ BMI & 45 ≤ age < 65)

− 2.1988 × (BMI = missing & 45 ≤ age < 65)

− 1.5956 × (BMI < 23 & age ≥ 65)

− 1.6144 × (23 ≤ BMI < 25 & age ≥ 65)

 − 1.9830 × (25 ≤ BMI < 30 & age ≥ 65)

− 2.2148 × (30 ≤ BMI < 35 & age ≥ 65)

− 2.6448 × (35 ≤ BMI & age ≥ 65)

− 2.4209 × (BMI = missing & age ≥ 65).

Scale = 0.8419

**m** = log(follow-up time in days) – μ

scale

**p** = 1- exp (-exp^m^)

**Number of diabetes cases** = p * survey weights

*Abbreviation: BMI, body mass index*

References

1. Rosella LC, Lebenbaum M, Li Y, Wang J, Manuel DG. Risk distribution and its influence on the population targets for diabetes prevention. *Preventive medicine.* 2014;58:17-21.

**Supplementary Table S2** Weighted baseline characteristics at survey date, Canada, by population group (Canada. 2017/18).

| **Population Group** | **1**  **SES low, Lifestyle low** | **2**  **SES low**  **Lifestyle high** | 3  **SES high**  **Lifestyle low** | 4  **SES high**  **Lifestyle high** |
| --- | --- | --- | --- | --- |
| **Overall** | n=6,348  8.1% | n=24,216  27.9% | n=7,920  11.4% | n=47,221  53.5% |
| **Sex** |  |  |  |  |
| Female | 66.6 | 46.7 | 62.9 | 51.4 |
| Male | 33.4 | 53.3 | 37.1 | 48.6 |
| **Age group (years)** |  |  |  |  |
| Age ≤ 45 | 54.7 | 40.3 | 64.1 | 44.6 |
| 45 < Age < 65 | 34.0 | 42.5 | 25.1 | 33.5 |
| Age ≥ 65 | 11.3 | 17.1 | 10.8 | 21.9 |
| **Racialized population group** |  |  |  |  |
| Yes | 0.0 | 0.0 | 48.1 | 37.5 |
| No | 100.0 | 100.0 | 51.9 | 62.5 |
| **Household income quintile** |  |  |  |  |
| Lowest | 0.0 | 0.0 | 28.7 | 28.7 |
| Low-middle | 0.0 | 0.0 | 28.1 | 29.9 |
| Middle | 24.9 | 26.8 | 16.6 | 16.5 |
| High-middle | 32.6 | 33.7 | 13.1 | 14.0 |
| Highest | 42.4 | 39.5 | 13.4 | 10.9 |
| **Highest household education** |  |  |  |  |
| Less than secondary | 0.0 | 0.0 | 6.8 | 17.2 |
| Secondary graduation | 0.0 | 0.0 | 35.0 | 35.5 |
| Post-secondary | 100.0 | 100.0 | 58.1 | 47.3 |
| **Household food insecurity** |  |  |  |  |
| Severely food insecure | 0.0 | 0.0 | 2.8 | 3.9 |
| Moderately food insecure | 0.0 | 0.0 | 6.8 | 8.4 |
| Food secure | 99.8 | 99.7 | 89.6 | 86.9 |
| **Amount of physical activity (PA) in past week (METs*min/week)** |  |  |  |  |
| PA = 0 | 0.0 | 16.9 | 0.0 | 27.1 |
| 0 < PA < 450 | 0.0 | 24.3 | 0.0 | 26.2 |
| 450 ≤ PA < 900 | 27.2 | 16.6 | 29.8 | 12.7 |
| PA ≥ 900 | 70.0 | 40.7 | 66.2 | 31.7 |
| **BMI (Body mass index, kg/m^2^)** |  |  |  |  |
| BMI < 23 | 51.0 | 10.5 | 54.5 | 15.8 |
| 23 ≤ BMI < 25 | 42.6 | 9.8 | 37.8 | 11.2 |
| 25 ≤ BMI < 30 | 0.0 | 50.3 | 0.0 | 43.2 |
| 30 ≤ BMI < 35 | 0.0 | 18.8 | 0.0 | 17.4 |
| BMI ≥ 35 | 0.0 | 8.3 | 0.0 | 8.6 |
| **Hypertension** |  |  |  |  |
| Yes | 0.0 | 18.1 | 0.0 | 20.8 |
| No | 100.0 | 81.9 | 100.0 | 79.2 |
| **Current smoker** |  |  |  |  |
| Yes | 0.0 | 15.6 | 0.0 | 24.1 |
| No | 100.0 | 84.4 | 100.0 | 75.9 |

**Supplementary Table S3** Weighted baseline characteristics at survey date, Canada, by excluded individuals with missing predictor information required for DPoRT (n=4816) (Canada. 2017/18).

| **Characteristic** | **%** |
| --- | --- |
| **Overall** | n=4816 |
| **Sex** |  |
| Female | 33.1 |
| Male | 66.9 |
| **Age group (years)** |  |
| Age ≤ 45 | 38.8 |
| 45 < Age < 65 | 32.0 |
| Age ≥ 65 | 29.1 |
| **Racialized population group** |  |
| Yes | 18.8 |
| No | 51.5 |
| Missing | 29.7 |
| **Household income quintile** |  |
| Lowest | 25.5 |
| Low-middle | 25.2 |
| Middle | 21.0 |
| High-middle | 15.8 |
| Highest | 12.4 |
| Missing | 0.07 |
| **Highest household education** |  |
| Less than secondary | 12.5 |
| Secondary graduation | 16.6 |
| Post-secondary | 42.7 |
| Missing | 28.0 |
| **Household food insecurity** |  |
| Severely food insecure | 2.5 |
| Moderately food insecure | 5.2 |
| Food secure | 70.7 |
| Missing | 21.5 |
| **Amount of physical activity (PA) in past week (METs*min/week)** |  |
| PA = 0 | 31.0 |
| 0 < PA < 450 | 12.0 |
| 450 ≤ PA < 900 | 18.8 |
| PA ≥ 900 | 29.5 |
| Missing | 8.7 |
| **BMI (Body mass index, kg/m^2^)** |  |
| BMI < 23 | 14.1 |
| 23 ≤ BMI < 25 | 10.1 |
| 25 ≤ BMI < 30 | 22.9 |
| 30 ≤ BMI < 35 | 8.4 |
| BMI ≥ 35 | 4.3 |
| Missing | 40.2 |
| **Hypertension** |  |
| Yes | 20.3 |
| No | 74.9 |
| Missing | 4.8 |
| **Current smoker** |  |
| Yes | 18.8 |
| No | 77.7 |
| Missing | 3.4 |

**Supplementary Table S4** Sensitivity Analysis*. Baseline characteristics at survey date and DPoRT estimated 10-year diabetes risk and case estimates for Canada 2017/18 to 2027/2028.

|  | **Proportion of population (%)** | **10-year diabetes risk (%)** | **Number of new cases** **(1000s)** |
| --- | --- | --- | --- |
| **Overall**(n = 90,521,  represented population = 25,824,712) | 100.0% | 9.6% | 2470.0 |
| **Sex** |  |  |  |
| Female | 51.5% | 8.6% | 1147.8 |
| Male | 48.5% | 10.6% | 1322.2 |
| **Age group (years)** |  |  |  |
| Age ≤ 45 | 46.0% | 4.7% | 554.1 |
| 45 < Age < 65 | 34.9% | 13.2% | 1184.2 |
| Age ≥ 65 | 19.1% | 14.8% | 731.7 |
| **Province/Territory** |  |  |  |
| Newfoundland and Labrador | 1.5% | 9.3% | 35.2 |
| Prince Edward Island | 0.4% | 9.2% | 9.7 |
| Nova Scotia | 2.6% | 9.5% | 64.8 |
| New Brunswick | 2.0% | 9.3% | 48.3 |
| Quebec | 23.4% | 9.6% | 576.7 |
| Ontario | 38.8% | 9.5% | 956.3 |
| Manitoba | 3.3% | 9.5% | 81.9 |
| Saskatchewan | 2.9% | 9.4% | 70.1 |
| Alberta | 11.5% | 9.8% | 290.2 |
| British Columbia | 13.5% | 9.6% | 336.7 |
| **Racialized population group** |  |  |  |
| Yes | 25.1% | 11.7% | 760.7 |
| No | 74.9% | 8.8% | 1709.3 |
| **Household income quintile** |  |  |  |
| Lowest | 19.1% | 10.2% | 501.5 |
| Low-middle | 19.6% | 10.3% | 518.8 |
| Middle | 20.0% | 9.7% | 503.5 |
| High-middle | 20.4% | 9.4% | 495.1 |
| Highest | 20.9% | 8.3% | 450.4 |
| **Highest household education** |  |  |  |
| Less than secondary | 10.1% | 14.7% | 385.0 |
| Secondary graduation | 22.6% | 11.0% | 642.8 |
| Post-secondary | 65.5% | 8.2% | 1392.9 |
| **Household food insecurity** |  |  |  |
| Severely food insecure | 2.4% | 9.8% | 60.7 |
| Moderately food insecure | 5.2% | 10.3% | 139.1 |
| Food secure | 90.4% | 9.5% | 2228.5 |
| **Amount of physical activity (PA) in past week (METs*min/week)** |  |  |  |
| PA = 0 | 19.7% | 12.0% | 613.1 |
| 0 < PA < 450 | 20.5% | 10.1% | 531.5 |
| 450 ≤ PA < 900 | 16.6% | 9.1% | 387.9 |
| PA ≥ 900 | 40.5% | 8.2% | 860.8 |
| **BMI (Body mass index, kg/m^2^)** |  |  |  |
| BMI < 23 | 21.2% | 3.0% | 165.5 |
| 23 ≤ BMI < 25 | 16.0% | 5.1% | 209.2 |
| 25 ≤ BMI < 30 | 37.9% | 9.4% | 918.4 |
| 30 ≤ BMI < 35 | 14.0% | 16.9% | 612.7 |
| BMI ≥ 35 | 6.7% | 23.6% | 407.9 |
| **Hypertension** |  |  |  |
| Yes | 16.3% | 20.1% | 845.4 |
| No | 84.7% | 7.5% | 1624.6 |
| **Current smoker** |  |  |  |
| Yes | 17.1% | 8.9% | 392.3 |
| No | 82.9% | 9.7% | 2077.8 |

*Sensitivity analysis using single conditional mean imputation to examine the impact of missing data.

**Supplementary Table S5.** Sensitivity Analysis*. Diabetes Population Risk Tool (DPoRT) 10-year risk reductions and cases averted for Canada 2017/18 to 2027/28 at baseline and with a hypothetical intervention scenario that applies a 5% diabetes relative risk reduction (Canada. 2017/18).

|  | **Health determinant domain** | | **10-year diabetes risk** | | | **Number of new diabetes cases (thousands)** | | |
| --- | --- | --- | --- | --- | --- | --- | --- | --- |
| **Group** | **SES^a^** | **Lifestyle** | **Baseline** | **5% relative risk reduction** | **Absolute risk difference from baseline** | **Baseline (1000s)** | **5% relative risk reduction** | **Cases averted (1000s)** |
| **Overall** | | | | | | | | |
| 1 | Low risk | Low risk | 5.5% | 5.3% | 0.2% | 316.8 | 301.0 | 15.8 |
| 2 | Low risk | High risk | 12.3% | 11.7% | 0.6% | 342.3 | 325.2 | 17.1 |
| 3 | High risk | Low risk | 7.4% | 7.1% | 0.3% | 666.3 | 632.9 | 33.4 |
| 4 | High risk | High risk | 15.8% | 15.0% | 0.8% | 1071.5 | 1018.0 | 53.5 |
| **Females** | | | | | | | | |
| 1 | Low risk | Low risk | 4.5% | 4.3% | 0.2% | 142.5 | 135.4 | 7.1 |
| 2 | Low risk | High risk | 10.5% | 10.0% | 0.5% | 130.6 | 124.1 | 6.5 |
| 3 | High risk | Low risk | 6.8% | 6.4% | 0.4% | 340.5 | 323.5 | 17.0 |
| 4 | High risk | High risk | 14.3% | 13.6% | 0.7% | 483.8 | 459.6 | 24.2 |
| **Males** | | | | | | | | |
| 1 | Low risk | Low risk | 6.7% | 6.4% | 0.3% | 174.4 | 165.7 | 8.7 |
| 2 | Low risk | High risk | 13.8% | 13.1% | 0.7% | 211.7 | 201.2 | 10.5 |
| 3 | High risk | Low risk | 8.2% | 7.8% | 0.4% | 325.8 | 309.5 | 16.3 |
| 4 | High risk | High risk | 17.4% | 16.6% | 0.8% | 587.8 | 558.4 | 29.4 |

*^a^ SES: socioeconomic/structural.*

*^*^ Sensitivity analysis: Interventions were applied to population group 2 (at least two high-risk factors in lifestyle domain* and 0 risk factors in the socioeconomic/structural domain*), group 3 (*≤1 high risk factor in the lifestyle domain and *at least one high-risk factor in socioeconomic/structural domain), or group 4 (at least two high-risk factors in lifestyle domain and at least one high risk factor in socioeconomic/structural domain). Group 1, 2, 3, and 4 represent 23.6%, 11.5%, 37.0% and 27.9% of the population, respectively.*

**Supplementary Table S6** Sensitivity Analysis*. Diabetes Population Risk Tool (DPoRT) risk estimates and inequity across income quintiles and household education levels for Canada 2017/18 to 2027/28, before and after an intervention that results in a 5% relative risk reduction (Canada. 2017/18).

| **Income quintile** | **Baseline** | **After intervention** | | |
| --- | --- | --- | --- | --- |
|  |  | **Target population group: 2** | **Target population group: 3** | **Target population group: 4** |
| Lowest (1) | 10.5% | 10.5% | 10.3% | 10.2% |
| Low-middle (2) | 10.6% | 10.6% | 10.4% | 10.2% |
| Middle (3) | 10.1% | 10.0% | 9.8% | 9.9% |
| High-middle (4) | 9.7% | 9.6% | 9.6% | 9.5% |
| Highest (5) | 8.7% | 8.6% | 8.6% | 8.6% |
| Inequity Q5−Q1 (absolute)^a^ | 1.8% | 1.9% | 1.7% | 1.6% |
| Inequity (relative)^b^ | 17.0 | 18.0 | 16.5 | 15.7 |
| **Household education** | **Baseline** | **Target population group: 2** | **Target population group: 3** | **Target population group: 4** |
| Less than secondary (1) | 15.2% | 15.2% | 15.0% | 14.7% |
| Secondary graduation (2) | 11.5% | 11.5% | 11.2% | 11.1% |
| Post-secondary (3) | 8.6% | 8.4% | 8.5% | 8.4% |
| Inequity 1−3 (absolute)^a^ | 6.6% | 6.8% | 6.5% | 6.3% |
| Inequity (relative)^b^ | 43.4 | 44.7 | 43.3 | 42.9 |

*^a^ Absolute inequity: risk difference between the most and least disadvantaged groups.*

*^b^ Relative inequity: risk difference between the most and least disadvantaged groups, divided by the risk of the most disadvantaged group.*

*^*^ Sensitivity analysis: Interventions were applied to population group 2 (at least two high-risk factors in lifestyle domain* and 0 risk factors in the socioeconomic/structural domain*), group 3 (*≤1 high risk factor in the lifestyle domain and *at least one high-risk factor in socioeconomic/structural domain), or group 4 (at least two high-risk factors in lifestyle domain and at least one high risk factor in socioeconomic/structural domain). Group 1, 2, 3, and 4 represent 23.6%, 11.5%, 37.0% and 27.9% of the population, respectively.*
